# A Novel Injectable Polypeptide Nanoparticle Encapsulated siRNA Targeting TGF‐β1 and COX‐2 for Localized Fat Reduction II: Phase I Clinical Trial

**DOI:** 10.1111/jocd.16722

**Published:** 2024-12-18

**Authors:** Mark S. Nestor, John Hetzel, Nardin Awad, Vishnu Bhupalam, Patrick Lu, Michael Molyneaux

**Affiliations:** ^1^ Center for Clinical and Cosmetic Research Aventura Florida USA; ^2^ Department of Dermatology and Cutaneous Surgery, University of Miami Miller School of Medicine Miami Florida USA; ^3^ Sirnaomics Inc. Gaithersburg Maryland USA

**Keywords:** adipose, clinical trial, COX‐2, fat, siRNA, TGF‐β1

## Abstract

**Background:**

Rising demand for non‐invasive body contouring is driven by aesthetics and the obesity epidemic. Deoxycholic acid (DCA) is the only FDA‐approved injectable for fat reduction but can cause side effects and significant local skin reactions (LSR). RNA interference, using small interfering RNA (siRNA) molecules, offers targeted fat reduction by silencing genes involved in fat maintenance. STP705, a siRNA injectable targeting TGF‐β1 and COX‐2, has shown promising preclinical results both in vitro and in animal models.

**Aims:**

To evaluate the safety and tolerability of STP705 for localized fat reduction in subjects undergoing abdominoplasty.

**Methods:**

This phase I dose‐ranging, randomized, vehicle‐controlled trial involved eight females undergoing abdominoplasty who received subcutaneous STP705 injections at varying concentrations and volumes in designated abdominal zones. Safety assessments, including physical exams, lab tests, ECGs, and local skin reactions (LSRs), were conducted at baseline and follow‐ups. Histopathologic evaluations of biopsies collected during abdominoplasty assessed adipocyte apoptosis and tissue remodeling.

**Results:**

STP705 demonstrated a favorable safety profile with no clinically significant changes in lab values, vital signs, or ECGs. Adverse events (AEs) were rare and transient. The incidence, intensity, and duration of LSRs were low throughout the study. Histological analysis revealed adipocyte destruction, fat remodeling, and necrosis.

**Conclusion:**

STP705 was safe and very well‐tolerated and showed preliminary efficacy in inducing adipocyte apoptosis and tissue remodeling, suggesting a safer alternative or adjunct to existing fat reduction therapies. These findings support further trials to establish the safety and efficacy of STP705 for targeted fat reduction and body contouring.

**Trial Registration:**

ClinicalTrials.gov identifier: NCT05422378

## 1 Introduction

Interest in body contouring and fat reduction procedures is increasing in the United States and across the world as a heightening focus on physical aesthetics along with the ongoing epidemic of obesity [1]. Relatively recent innovations in non‐invasive methods for body contouring, such as energy‐based devices and injectable agents, have helped to meet a rise in demand while allowing for a more individual and tailored treatment approach. Deoxycholic acid (DCA), the only FDA‐approved injectable fat reduction agent, has shown good efficacy in treating submental fat deposits however frequently involves significant patient downtime and associated side effects like local pain, redness, and inflammatory reactions [2, 3]. Given that patients may not be willing to tolerate these risks and side effects, it is important to explore potential alternatives in order to optimize efficacy and side effects so that more patients are able to pursue their aesthetic goals.

Therapeutics based on the principle of RNA interference (RNAi) are rapidly developing and have broad potential for highly targeted treatments, including fat reduction [4]. These agents include small interfering RNA (siRNA) molecules that complement specific messenger RNAs whose genes have key links to cellular processes including tumor and disease pathogenesis [4, 5, 6]. During RNAi, siRNA agents bind to target mRNA molecules and trigger their degradation, thereby effectively silencing the genes from which they originated [7]. In the case of fat reduction, evidence indicates that adipocyte populations in stubborn fat deposits may be maintained partly through TGF‐β1/Smad3 and COX‐2 signaling pathways, which are strongly implicated in obesity and type 2 diabetes. Silencing the expression of TGF‐β1 and COX‐2 in the adipocytes of stubborn fat may significantly impair their metabolic function and trigger apoptosis, thereby achieving targeted fat reduction [8, 9, 10, 11, 12]. Preclinical in vitro and animal model studies of an injectable agent consisting of TGF‐β1 and COX‐2 siRNAs encapsulated in a polypeptide nanoparticle (PNP) vehicle, have demonstrated its safety and tolerability, substantiated its mechanisms, and provided preliminary evidence of efficacy for fat reduction which appeared to be at least equal to that of DCA [13]. The aim of this phase I clinical trial was to assess the safety and tolerability of STP705 injections in subjects undergoing planned abdominoplasty. A secondary aim was to make initial histologic observations on the efficacy of STP705 injections in inducing adipocyte apoptosis and tissue remodeling for the purpose of targeted fat reduction and body contouring.

## 2 Methods

### 2.1 Study Design and Objectives

This was a phase I, dose‐ranging, randomized, vehicle‐controlled study to evaluate the safety and tolerability of subcutaneous injection of STP705 in adult subjects (age 18–65) undergoing scheduled abdominoplasty upon study completion. It was IRB approved (WCG IRB, Miami Florida, Study Number: 1331009) and listed on Clinical Trials.gov (ClinicalTrials.gov ID NCT05422378). Ten subjects met inclusion/exclusion criteria, and eight subjects were enrolled and completed the study per protocol. A specific number of subjects were chosen to best assess the safety of multiple dosing regimes for this phase I trial.

#### 2.1.1 Objectives

1. To assess injection comfort, characterize local and systemic safety, and evaluate histological changes for subcutaneous doses of STP705.
2. To compare the safety and tolerability of three different concentrations of STP705 in two different injection volumes each in order to select safe and tolerable dose(s) of STP705 for subsequent study.

For each subject, the central to lower abdominal region designated for future abdominoplasty was divided into three distinct injection zones, all below the level of the umbilicus. Each injection zone was subdivided into 1–3 injection sites designated by the investigator for a total of seven injection sites. An unblinded pharmacist randomly assigned each of the seven treatments described in Table 1 to 1 of the seven injection sites, which were subsequently tattooed with an identifier to ensure accurate treatment administration.

**TABLE 1:** Treatments administered.

Treatment	Test article	Volume of injection
1	STP705, 120 μg/mL	0.5 cc
2	STP705, 120 μg/mL	1.0 cc
3	STP705, 240 μg/mL	0.5 cc
4	STP705, 240 μg/mL	1.0 cc
5	STP705, 320 μg/mL	0.5 cc
6	STP705, 320 μg/mL	1.0 cc
7	Vehicle	1.0 cc

Injections of the specified doses of STP705 or placebo were administered to their assigned sites on Day 1/Baseline. Subsequent injections were completed in the same manner on Day 29 and Day 57, pending a favorable review of local skin reactions (LSRs). Various safety measurements were performed at baseline and during scheduled follow‐ups occurring at 2 and 7 days, post‐procedure. Safety measurements included physical examination, vital signs, clinical laboratory tests, urine pregnancy tests (UPTs), 12‐lead electrocardiograms (ECGs), injection site ultrasounds, injection site LSRs, and recording of adverse events (AEs). Histopathologic evaluation of injection site biopsies and evaluations of local fat reduction were used to assess treatment efficacy.

After the completion of day 85 (28 days after the last injection procedure), each subject underwent an abdominoplasty procedure per the standard of care. The subject consented to the abdominoplasty procedure per the surgeon's site practices. Injection site biopsies were collected from the tissue removed during the abdominoplasty procedure and subsequently used for histopathologic evaluations.

### 2.2 Composition of the Study Agent

STP705 (Sirnaomics) is composed of 2 siRNA oligonucleotides, pixofisiran and lixadesiran, that individually target TGF‐β1 and COX‐2 mRNA, respectively. The siRNA elements of the study agent are encapsulated in a polypeptide nanoparticle (PNP) vehicle (siRNA:peptide mass ratio of 1:2.5) consisting of a histidine‐lysine co‐polymer (HKP). The resulting product is formulated as a lyophilized powder that is reconstituted with sterile water prior to injection.

### 2.3 Local Skin Reactions

Investigator‐assessed LSRs (erythema, edema, and bruising) and subject‐assessed LSRs (pain and stinging/burning) were each evaluated using a 4‐point ordinal scale (0 = absent to 3 = severe). Assessments were completed prior to and after each treatment procedure. For these LSRs, only those that required medical intervention (e.g., prescription medication), treatment modification, or discontinuation of treatment were documented as AEs.

### 2.4 Histopathologic Evaluation of Abdominoplasty Specimens

Excisional biopsies consisting of 2 × 2 cm tissue samples centered on the injection site were taken from each of the injection sites during the abdominoplasty procedures following treatment completion. Samples were processed by a blinded dermatopathologist and analyzed for fat necrosis, lipolysis, foreign body giant cell reaction, vasculopathic changes, and acute inflammation. A histologic score indicating the degree of fat apoptosis, necrosis, and inflammatory response was assigned to each sample based on the following scale: no change = 0; mild inflammation and/or mild fibrosis = 1; lymphocytic infiltration and hemorrhage = 2; panniculitis and fibrosis = 3; panniculitis and lymphocytic infiltration = 4; panniculitis and fat necrosis = 5.

### 2.5 Statistical Analysis

All statistical analyses were performed using SAS, version 9.4 or higher. No formal tests of hypotheses were planned or performed. Histological scores for each dosage were compared using a Kruskal‐Wallis test; subsequent pairwise assessment was performed using Dunn's multiple comparisons test.

## 3 Results

Overall, there were no material safety issues identified for STP705 based on safety assessments conducted during the study. There were no clinically significant changes in baseline lab values, vital signs, or ECGs. Across 728 total injections, only three moderate AEs (edema, erythema, and pruritis) all at one injection site, at one injection cycle, in one subject deemed likely to be related to STP705 were observed and were treated with hydrocortisone cream, and rapidly resolved without dose modification. The incidence, intensity, and duration of LSRs were low throughout the study. Histological evaluation showed adipocyte destruction and fat remodeling across most dosing parameters. Overall, STP705 was well tolerated by the study subjects at each of the tested concentrations and volumes.

### 3.1 Adverse Events

Over the course of the study, with a total of 728 potential evaluable local injection‐related AE's, only three moderate AEs deemed likely to be related to STP705 were observed, and as noted, they were in a single subject in one injection site at one visit (1.0 cc of STP705 240 μg/mL treatment area on Day 65, 8 days after the last dose of STP705) and resolved without dose modification or other deviations in the study protocol. The reaction was characterized by edema, erythema, and pruritus; each of these was reported as an AE and deemed likely to be related to the treatment. The affected area was treated with OTC 1% hydrocortisone cream and resolved without sequelae on Day 73 (duration of 8 days). There were no systemic AEs reported during the study.

### 3.2 Local Skin Reactions

Erythema was the most common local skin reaction (LSR), occurring in 32.3% of evaluations of injection sites (235/728). Most cases of erythema were mild (221/235), with moderate cases noted in very few injection sites with a trend to higher doses of STP 705 (14/235). 15.3% of vehicle‐treated areas reported erythema as well (16/104), and no severe erythema in any of the injection sites occurred during the study. The overall mean duration of erythema when observed was less than 6 days (Table 2).

**TABLE 2:** Overall and severity‐stratified incidence of the local skin reactions.

LSR type	Overall incidence	Mild cases	Moderate cases	Severe cases	Mean duration (days)	Specific details
Erythema	32.3% (235/728)	30.4% (221/728)	1.9% (14/728)	None	< 6	Most common in higher STP705 concentrations; minimal in vehicle areas (16/104)
Edema	8.3% (61/728)	6.8% (50/728)	1.5% (11/728)	None	~3	1.0 cc of 320 μg/mL STP705 area: most edema cases (22/728), with moderate cases only occurring at this dose
Bruising	5.9% (43/728)	4.5% (33/728)	1.4% (10/728)	None	~10	Incidence and severity were similar across all treatment areas
Pain	1.5% (11/728)	0.7% (5/728)	0.4% (3/728)	0.4% (3/728)	~1	Minimal in vehicle areas: 1 moderate, 2 mild reports (0.4%)
Burning/Stinging	3.2% (23/728)	2.1% (15/728)	0.8% (6/728)	0.3% (2/728)	~1.5	STP705 120 μg/mL: Most reports (12/728; 1.6%) and highest severity (4 moderate, 2 severe)

Edema was reported in 8.3% of evaluations of injection sites (61/728), and most of these cases were mild (50/61). The treatment areas injected with 1.0 cc of STP705 320 μg/mL had the most reports of edema (22/61) as well as the only reports of moderate injection site edema (11/61). The overall mean duration of edema, when observed, was approximately 3 days.

**FIGURE 1:**
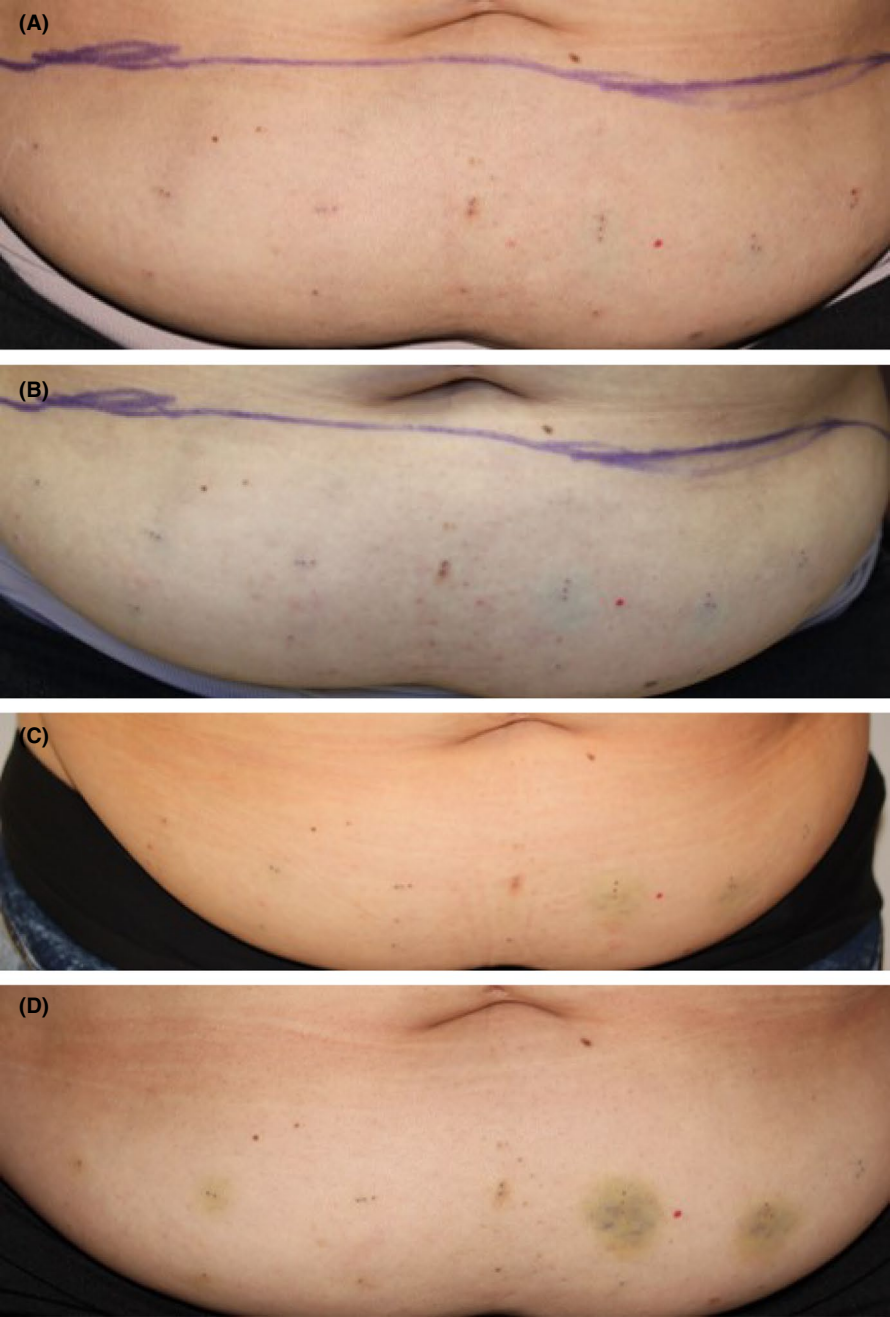
Photographic example of injection sites. (A) Pre‐treatment. (B) Post‐treatment. (C) Post‐treatment Day 3. (D) Post‐treatment Day 8.

Bruising was minimal, noted in 5.9% of evaluations of injection sites (43/728), with similar incidence and severity across all injection sites, including vehicle and was thought to be from the injection procedure rather than the investigational product (IP). Most were mild (33/43), with some moderate bruising (10/43). The overall mean duration of bruising, when observed, was approximately 10 days. Figure 1 shows an example of LSRs at baseline, immediately post‐treatment, and days 3 and 8 post‐treatment.

Pain was reported in only 1.5% of injection sites (11/728). Most pain cases were mild (5/11), with a few moderate (3/11) and severe (3/11) instances. Pain reports did not appear to be associated with either injected dose or volume. The vehicle‐treated areas recorded one moderate and two mild pain reports (3/11). No pain was reported after the third treatment. The overall mean duration of pain, when reported, was approximately 1 day.

Burning/stinging was relatively uncommon and reported in 3.2% of injections (23/728), mostly mild (15/23) with few moderate (6/23) and severe (2/23) cases. Burning/stinging was not associated with the injected dose or volume, with 120 μg/mL STP705 concentration having the most reports (12/23) and the highest severity (4 moderate and 2 severe). The overall mean duration of burning/stinging, when reported, was approximately 1.5 days.

### 3.3 Histological Analysis

**FIGURE 2:**
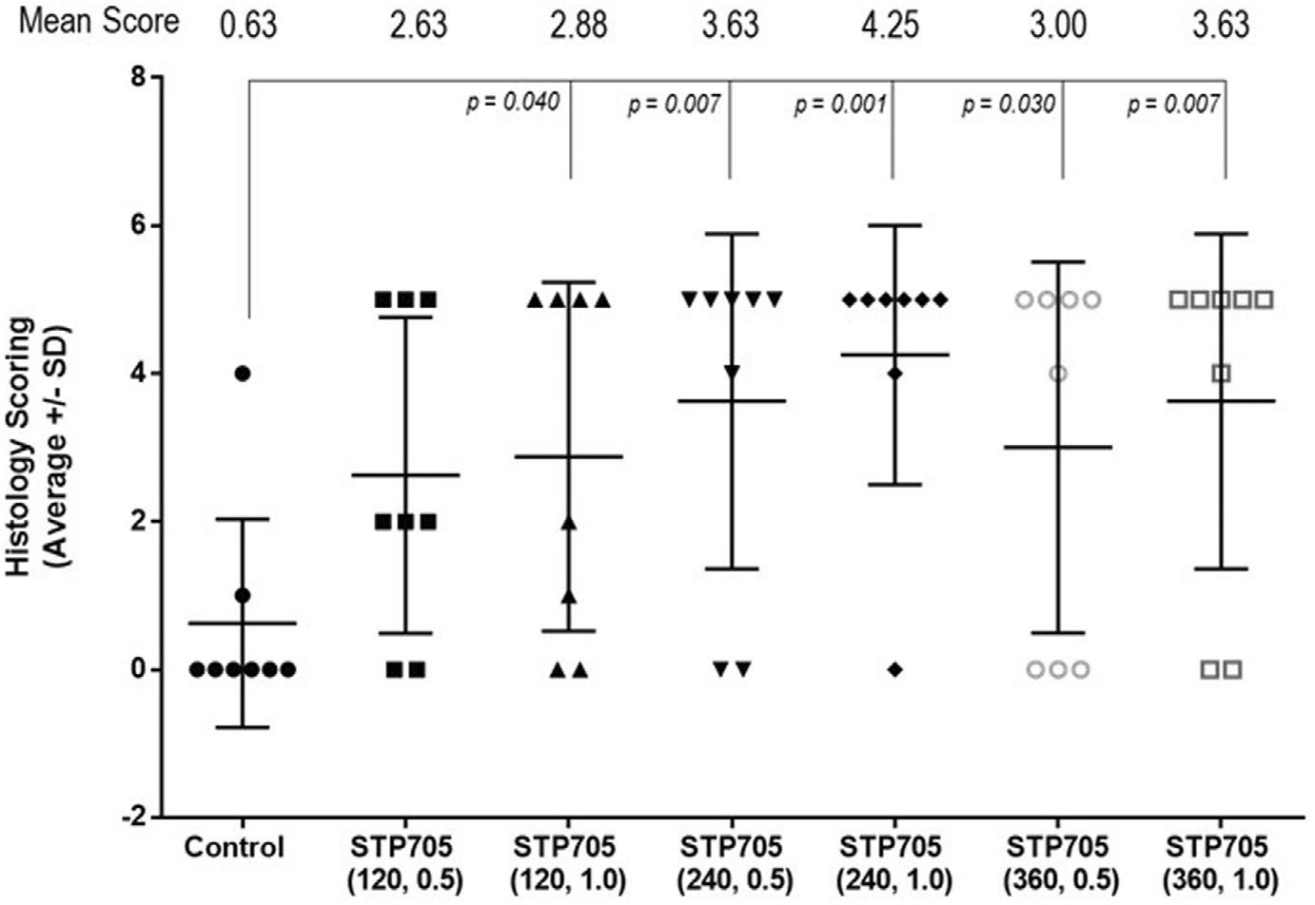
Summary of histology scoring.

**FIGURE 3:**
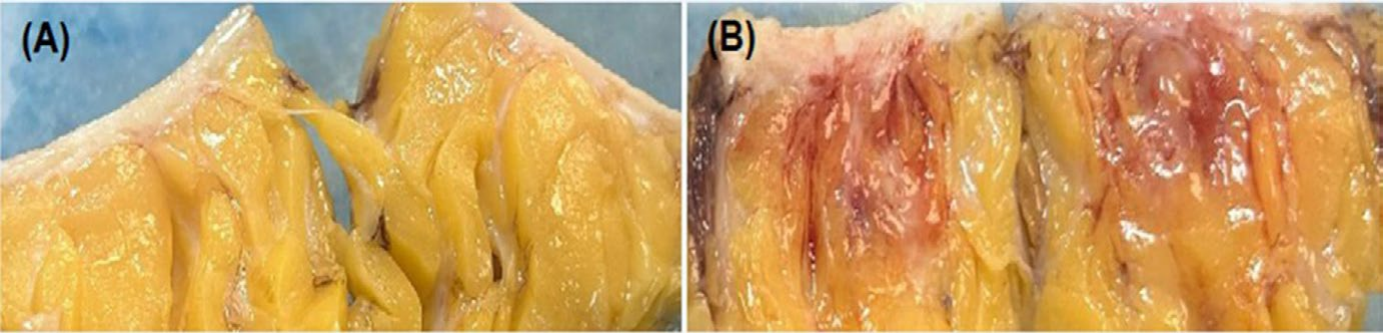
Excisional adipose tissue samples. (A) Site with placebo injection demonstrating normal tissue appearance. (B) Site with 240 μg STP705 in a 1.0 mL injection volume. The sample displays gross evidence of adipocyte necrosis at the injection sites.

Varying degrees of inflammation, lipolysis, and fat necrosis were observed in the treatment areas injected with STP705. Injected areas receiving 240 μg/mL STP705 had the highest incidence of fat necrosis (10/16 [62.5%] specimens), and a difference was also observed at higher dosages (320 μg/mL, 8/16 [50%] specimens). No fat necrosis was present in treatment areas injected with only the vehicle. There was notable inter‐subject variability in tissue response to STP705, with some subjects having fat necrosis in all treated areas (excluding those areas injected with the vehicle only). Figure 2 displays the mean (±SD) histological scoring of the excised tissue samples. Each of the treatments (excluding STP705 120 μg/mL 0.5 cc) was associated with a statistically significantly higher histology score as compared to the vehicle (control). The 240 μg/mL, 1.0 cc dose had the highest score 4.25 versus. the vehicle 0.63 (*p* = 0.001). Figure 3 shows gross evidence of adipocyte necrosis in tissue treated with STP705; by comparison, the tissue treated with only the vehicle does not show evidence of necrosis.

## 4 Discussion

There is a rising interest in non‐invasive body contouring and fat reduction procedures, a phenomenon that may partly be a response to an increasing societal focus on physical aesthetics coupled with the growing prevalence of obesity [1]. Among the technologies developed to meet the demand for targeted fat reduction, injectable DCA has gained popularity due to its efficacy, and minimally invasive nature [2, 14]. However, despite these benefits, DCA treatments carry meaningful risks, adverse events, and significant LSR, including significant patient downtime and a range of unpleasant side effects like pain, redness, and inflammatory reactions of meaningful duration [2]. These side effects may represent a barrier to treatment for potential patients, so it is worthwhile to investigate therapeutic alternatives that are safer yet still effective, thereby broadening the appeal and accessibility of fat reduction procedures. This phase I clinical trial assessed the safety and tolerability of STP705, an innovative injectable siRNA therapy targeting TGF‐β1 and COX‐2 for localized fat reduction. The results demonstrate that STP705 has a favorable safety profile and also provide preliminary insights into its efficacy while building upon the in vitro and animal data [13].

Throughout the study, STP705 was shown to be well‐tolerated at all included concentrations and volumes. LSRs, such as erythema, edema, and bruising, were closely monitored and had a low incidence across all treatment groups. Furthermore, these reactions were mostly mild and transient, drawing a contrast with those typically elicited by DCA, which often result in considerable discomfort and patient downtime. Erythema, edema, and bruising were the most commonly reported LSRs, and there were no reports of severe cases for these reactions. Reports of pain and stinging/burning were even more infrequent and generally mild. Nearly all of the reported local reactions were resolved without any intervention within just a few days of the injection procedure. As with LSRs, IP‐related AEs were rare throughout the study. All of the three reported AEs occurred in a single injection site on one patient, and they also resolved within days without significant interventions beyond the use of topical hydrocortisone. Comprehensive safety assessments yielded no clinically significant changes from baseline in any of the patients, confirming that STP705 treatments have minimal systemic effect. These findings evidence the excellent safety and tolerability of STP705 injections and set the stage for future human trials on a larger scale.

Preclinical studies of STP705 previously demonstrated its capacity to silence TGF‐β1 and COX‐2, which are thought to be key mediators in the development and maintenance of stubborn fat deposits. TGF‐β1 is a cytokine essential for cellular growth, differentiation, and inflammation. It promotes the transformation of preadipocytes into mature fat cells and contributes to obesity‐related fat accumulation by enhancing fibrotic responses in adipose tissues, resulting in less active fat depots [13, 15]. COX‐2, an important inflammation‐associated enzyme, affects prostaglandin production in adipose tissues and plays a part in regulating adipocyte development. Elevated COX‐2 levels are associated with increased fat deposition and chronic adipose inflammation, affecting overall metabolic activity in fat cells [8, 9, 10, 11, 12]. By disrupting the TGF‐β1 and COX‐2 signaling pathways simultaneously, STP705 significantly impairs adipocyte metabolic functions and can induce adipocyte apoptosis, leading to fat reduction [11, 12]. Preclinical porcine studies of STP705 have shown its effectiveness in reducing the thickness of subcutaneous tissue, which appeared to be at least equal to that of DCA [13]. The results of the histological analysis from this investigation give early insights into the effectiveness of STP705 injections for fat reduction in human subjects, and the observed marginally dose‐dependent effect will help to guide optimal dosing parameters for future large‐scale investigations.

A limitation of this phase I study is that it included a relatively small number of subjects although there was a total of over 750 injection sites that were assessed. Based on the data from this study, a more robust phase I/IIA study will narrow the treatment range to two or three doses and include a safety assessment of the submental area.

## 5 Conclusion

In summary, this phase I clinical trial provides evidence establishing the excellent safety and tolerability of STP705 in humans while also providing a preliminary confirmation of the promising efficacy for fat reduction observed in preclinical porcine models. These findings suggest that treatment with STP705 could be useful as an alternative to DCA or as an adjunct therapy used in combination with other methods for fat reduction. Though this study was somewhat limited by its small size and short follow‐up, the results still provide a strong justification for future clinical trials to further establish the safety and efficacy of STP705 injections for targeted fat reduction and body contouring.

## Author Contributions

All authors met journal contribution guidelines.

## Disclosure

Dr. Nestor is a consultant for Sirnaomics, and Dr.'s Lu and Molyneaux are employed by Sirnaomics.

## Ethics Statement

The authors confirm that the ethical policies of the journal, as noted on the journal's author guidelines page, have been adhered to IRB approved (WCG IRB, Miami Florida, Study Number: 1331009).

## Conflicts of Interest

Dr. Nestor is a consultant to Sirnaomics Inc. and Dr's Lu and Molyneaux are employees of Sirnaomics.

## Data Availability

The data that support the findings of this study are available from the corresponding author upon reasonable request.
